# Role of MIF gene polymorphisms in systemic lupus erythematosus and prospects for therapeutic intervention

**DOI:** 10.1186/ar3967

**Published:** 2012-09-27

**Authors:** R Bucala

**Affiliations:** 1 Yale School of Medicine, New Haven, CT, USA

## 

The cytokine macrophage migration inhibitory factor (MIF) counter-regulates the immunosuppressive action of glucocorticoids and inhibits activation-induced apoptosis. MIF is encoded in a polymorphic locus with a variant allele frequency >5% and high-expression alleles are associated with clinical severity of rheumatoid arthritis, scleroderma, and asthma but improved outcome from pneumonia.

We completed a candidate gene association study in SLE (3195 patients and controls) to examine the relationship between two promoter polymorphisms in *MIF*: a -794 CATT_5-8 _microsatellite repeat (*rs5844572*), where increased repeat number leads to greater *MIF *expression; and a -173 G/C SNP (*rs755622*) that is in linkage disequilibrium with CATT_7_. Patients with the high-expression CATT_7 _allele had lower SLE incidence: OR = 0.63, *P *= 0.001 in Caucasians and OR = 0.46, *P *= 0.012 in African-Americans. Among patients with established SLE, those with serositis, nephritis, and cerebritis had reduced frequencies of low-expression *MIF *genotypes (CATT_5_) when compared with patients without end-organ involvement (*P *= 0.005 for serositis, *P *= 0.023 for nephritis, and *P *= 0.04 for cerebritis). Plasma MIF levels and TLR4, TLR7, and TLR9 stimulated MIF production reflected the underlying *MIF *genotype of the studied groups. These data suggest that MIF exerts a dual influence on the immunopathogenesis of SLE. High-expression *MIF *genotypes are associated with a reduced susceptibility to SLE, perhaps by contributing to enhanced clearance of infectious agents. Once SLE develops, however, low-expression *MIF *genotypes may protect from ensuing inflammatory end-organ damage [1].

We also examined the therapeutic impact of MIF antagonism in two mouse models of spontaneous SLE. In both the NZB/NZW F1 and the MRL/*lpr *mouse strains, anti-MIF or small molecule MIF receptor antagonism reduced functional and histological indices of glomerulonephritis, MIF-R^+ ^leukocyte recruitment, and proinflammatory cytokine and chemokine expression [2]. A humanized anti-MIF developed from our studies has recently entered phase I clinical testing [3].

These data highlight the importance of MIF in the development of autoimmunity and support the potential clinical feasibility of pharmacologic MIF antagonism. We anticipate that MIF antagonists may be most effectively applied in those individuals who, on the basis of their genotype, manifest a MIF-dependent form of autoimmunity.
